# Extracellular vesicles in mycobacteria: new findings in biogenesis, host-pathogen interactions, and diagnostics

**DOI:** 10.1128/mbio.02552-23

**Published:** 2024-04-03

**Authors:** Vivian C. Salgueiro, Charlotte Passemar, Lucía Vázquez-Iniesta, Laura Lerma, Andrés Floto, Rafael Prados-Rosales

**Affiliations:** 1 Department of Preventive Medicine, Public Health, and Microbiology. School of Medicine, Universidad Autónoma de Madrid, Madrid, Spain; 2 Cambridge Center for Lung Infection, Royal Papworth Hospital NHS Trust, Cambridge, United Kingdom; Instituto Carlos Chagas, Curitiba, Brazil

**Keywords:** *Mycobacterium tuberculosis*, biogenesis, extracellular vesicles

## Abstract

Since the discovery of extracellular vesicles (EVs) in mycobacterial species 15 years back, we have learned that this phenomenon is conserved in the *Mycobacterium* genus and has critical roles in bacterial physiology and host-pathogen interactions. *Mycobacterium tuberculosis* (*Mtb*), the tuberculosis (TB) causative agent, produces EVs both *in vitro* and *in vivo* including a diverse set of biomolecules with demonstrated immunomodulatory effects. Moreover, *Mtb* EVs (MEVs) have been shown to possess vaccine properties and carry biomarkers with diagnostic capacity. Although information on MEV biogenesis relative to other bacterial species is scarce, recent studies have shed light on how MEVs originate and are released to the extracellular space. In this minireview, we discuss past and new information about the vesiculogenesis phenomenon in *Mtb*, including biogenesis, MEV cargo, aspects in the context of host-pathogen interactions, and applications that could help to develop effective tools to tackle the disease.

## INTRODUCTION

*Mycobacterium tuberculosis* (*Mtb*), the causative agent of tuberculosis (TB), has been the subject of intense investigation since its discovery by Robert Koch in 1882. Paradoxically, TB still represents one of the first causes of death by a single infectious agent worldwide according to the WHO TB report, with 1.3 million deaths in 2022 (1). Although there has been a significant recovery of TB diagnosis to pre-pandemic levels, the number of people infected with TB remains unacceptably high (7.5 million people were newly diagnosed with TB in 2022). Currently, TB treatment requires a 6-month regimen of four first-line drugs, which are ineffective in treating infections with multidrug-resistant (MDR) strains. Preventative vaccination against TB employs a century-old, attenuated *Mycobacterium bovis* strain (Bacillus Calmette-Guérin or BCG) that does not offer protection after adolescence. Thus, TB remains one of the greatest global health threats and there is an urgent need to develop effective tools to strengthen its control. *Mtb* is an intracellular and metabolically flexible bacterium that has successfully spread in the human population due to a superb adaptation to environmental changes through the course of infection, including oxygen and nutrient deprivation, and stresses imposed by the intraphagosomal environment within phagocytic cells (2). Humans serve as the only known natural host and reservoir for *Mtb*, in large part due to exquisite mechanisms of immunomodulation and immune evasion developed by *Mtb* that allow it to survive within immune cells that are usually able to contain or eradicate most bacteria.

Like other intracellular pathogens, *Mtb* secretes proteins and lipids that play key roles in survival and persistence while it is confined within phagocytes (3). *Mtb* accomplishes this using general (Tat and Sec) and specialized (Type VII) secretion systems. In addition, similar to most forms of life, *Mtb* can release antigens using an alternative mechanism based on extracellular vesicles (EVs) (4), recently named the Type 0 secretion system (5). Since the first detection of bacterial EVs in *Escherichia coli* more than 60 years ago (6), subsequent studies have demonstrated their functional commonality in many bacterial species.

We previously demonstrated that *Mtb* produces EVs *in vitro* and *in vivo* as part of a sophisticated mechanism to manipulate host cellular physiology and evade the host immune system (4, 7). *Mtb* EVs (MEVs) have immunomodulatory properties *in vitro* and when administered to mice (4, 8), have promising vaccine properties (9), and are a good source of biomarkers in serology studies (10, 11). General biogenesis mechanisms indicate that bacterial EVs can be produced naturally or induced by distinct stressors, and several studies support the notion that vesiculogenesis is genetically regulated (5, 12–14). Our group reported *virR* (vesiculogenesis and immune response regulator) as the first gene implicated in the biogenesis of EVs in *Mtb* (15, 16). A transposon mutant in *virR* overproduces MEVs, which provokes augmentation of cytokine responses in mouse and human macrophages. This mutant manifested an attenuated phenotype in experimental macrophage and mouse infections (15, 17). These studies have established a connection between vesiculogenesis and virulence. Since then, conditions such as iron starvation (18), the bacterial dynamin-like proteins IniA and IniC (19), and the Pst/SenX3-RegX3 signal transduction system (20) have been shown to contribute to the biogenesis of MEVs.

Consequently, MEVs have generated considerable interest for their potential role in TB pathogenesis and implications in the development of new preventive and therapeutic antitubercular strategies. This minireview provides context and illustrates the vesiculogenesis process in mycobacteria, with an emphasis on *M. tuberculosis* (Table 1).

**TABLE 1:** Timeline of landmark discoveries in *M. tuberculosis* extracellular vesicles

Year	Landmark	Reference(s)
2023	Bacterial dynamin-like proteins IniA and IniC contribute to the biogenesis of *Mtb* EVs	(19)
	*Mtb* vesiculogenesis may indirectly control cell permeability via PG remodeling	(16)
	VirR interacts with all LCP proteins via the LytR domain	(16)
2022	Isolated MEVs trigger intracellular toll-like receptor 8 (TLR-8) signaling *in vitro* and *in vivo*	(21)
2018	Pst/SenX3-RegX3 signal transduction system contributes to the biogenesis of *Mtb* EVs	(20)
2015	EVs in Gram-positive bacteria originate at the cell membrane and are known as cytoplasmic MVs	(22, 23)
2014	*Mtb* EVs administered to mice have promising vaccine properties	(9)
	*Mtb* increases EVs production in response to iron restriction	(24)
2013	*virR* as the first gene implicated in the biogenesis of *Mtb* EVs	(15, 16)
	Certain proteins enriched in *Mtb* EVs may constitute a novel TB biomarker signature	(10, 11)
2011	*Mtb* produces EVs with immunomodulatory properties *in vitro/in vivo*	(4, 8)
	*Mtb* EVs contribute to virulence and pathogenesis, helping to evade the host immune system	(4, 7)
2010	A role for Rv0431 (*virR*) in *Mtb* virulence	(17)
2007	First report of EVs in a mycobacterial species: *Mycobacterium ulcerans*	(25)
2006	Vesiculogenesis is genetically regulated in Gram-negative bacteria	(5, 12–14)
1990	First observation of EVs in Gram-positive bacteria	(26)
1989	Detection of DNA and RNA in EVs in Gram-negative bacteria	(27)
1965	First observation of EVs in Gram-negative bacteria	(6)

## BIOGENESIS OF MEVs

### Gram-negative and Gram-positive bacteria

**Fig 1:**
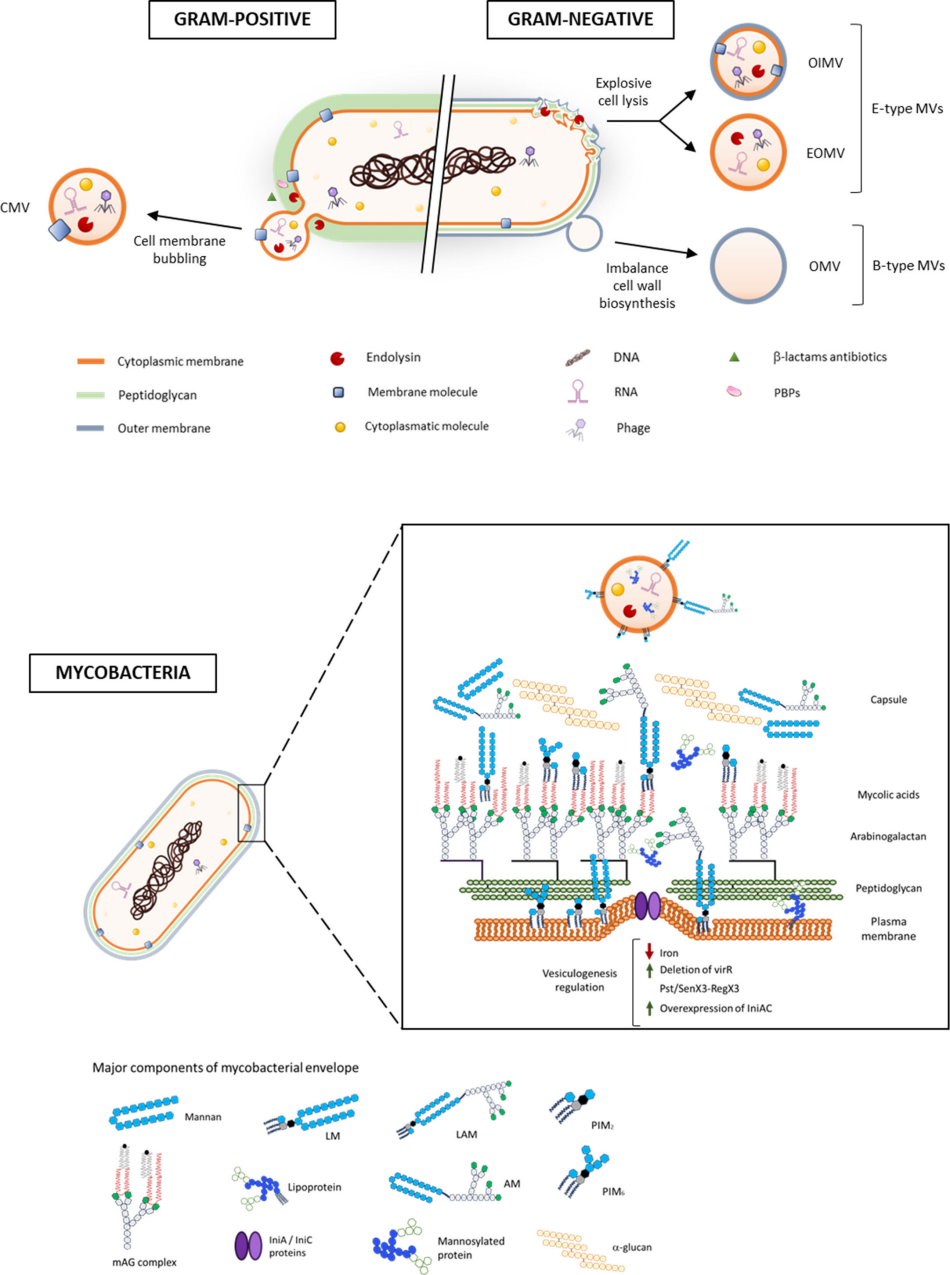
General and specific vesiculogenesis mechanisms in *Mtb*. In Gram-positive bacteria, EVs originate at the cell membrane giving CMVs through mechanisms involving cell wall modifications. In addition, chemical modifications via sublethal exposure to β-lactams or genetic deletion of PBPs, as well as the action of prophage endolysins, increase CMV release in a similar mechanism to that of Gram-negative explosive cell lysis. In Gram-negative bacteria, EVs are produced through outer membrane blebbing, forming OMVs (B-type MVs) or explosive cell lysis via prophage endolysins, forming OIMVs and EOMVs (E-type MVs). The composition of the EVs depends on the mechanism of biogenesis. In mycobacteria, MEVs are produced in the cell membrane and different factors influence their production, such as iron availability, *virR*, dynamin-like proteins IniAC, and the Pst/SenX3-RegX3 system. CMV, cytoplasmic membrane vesicle. OIMV, outer-inner membrane vesicle. EOMV, explosive outer membrane vesicle. OMV, outer membrane vesicle. MV, membrane vesicle. PBPs, penicillin-binding proteins. LM, lipomannan. LAM, lipoarabinomannan. PIM2, phosphatidylinositol dimannoside. PIM6, phosphatidylinositol hexamannoside. AM, arabinomannan. mAG complex, mycolyl arabinogalactan complex.

Since the first detection of bacterial EVs more than 60 years ago in cell-free supernatants of *Escherichia coli* (*E. coli*) cultures grown under lysine-limiting growth conditions (6), subsequent studies have demonstrated that vesiculation is a universal phenomenon in microorganisms. Bacterial EVs are defined as “spherical, membranous vesicles from microbial cell surfaces ranging in size from 20 to 500 nm in diameter” (22, 28). The biogenesis of EVs in bacteria depends strongly on the architecture of the cell envelope (Fig. 1). Thus, Gram-negative bacteria have two main routes of vesicle formation: (i) blebbing of the outer membrane (OM), forming outer membrane vesicles (OMVs) (also known as B-type EVs) (5). These OMVs encapsulate components solely from the periplasmic space before detaching and release to the extracellular space; (ii) alternatively, Gram-negative bacteria can generate EVs through a process termed explosive cell lysis, generating E-type EVs (5). Explosive cell lysis is mediated by the degradation of peptidoglycan (PG) fragments via prophages that are activated upon genotoxic stress. Recently, the binding of lytic phages to the OM has been shown to generate B-type EVs (29). E-type EVs are a diverse set of EVs whose composition depends on the components that integrate the particle and could include cytoplasmic, inner membrane, or outer membrane biomolecules.

The first EV study on a Gram-positive bacteria reported vesiculation in *Bacillus spp*. almost 30 years later than that of Gram-negative bacteria (26). The major hurdle in considering EV production by cell-walled microbes was the belief that the cell wall was a rigid structure that prevented vesicular transit. Today, we know that EVs in Gram-positive bacteria originate at the cell membrane and are known as cytoplasmic membrane vesicles (CMVs) (22, 23). In addition, several studies have demonstrated that cell wall modifications play a critical role in CMV release. In this context, chemical modification of PG via sublethal exposure to β-lactams or genetic deletion of penicillin-binding proteins (PBPs) has been shown to increase CMV release in *Staphylococcus aureus* (30), indicating that PG cross-linking plays a role in EV release. Interestingly, a mechanism similar to explosive cell lysis reported in Gram-negative bacteria has been observed in Gram-positive bacteria with some differences. Gram-negative bacteria membranes from dead cells round up and form E-type EVs, whereas Gram-positive bacteria preserve the morphology and EVs get to the extracellular space via holes in the PG (31).

### Mycobacteria

An explanation for how MEVs are released becomes more difficult for bacteria with unconventional cell envelopes, such as *Mtb*, since, besides the PG layer, MEVs must get through an additional envelope covalently linked to PG made of arabinogalactan (AG), which, in turn, is decorated with exceptionally long-chain (up to 60 Cs) mycolic acids (MA) that get intercalated by free lipids, constituting the mycomembrane (32) (Fig. 1). Therefore, the theoretically possible origin of MEVs could be either the cell membrane or the mycomembrane. Although the first study reporting the existence of EVs in mycobacterial species was undertaken in *Mycobacterium ulcerans*, no lipid analysis was performed (25). Subsequent studies on *Mtb* indicated that MEVs isolated from a minimal medium (MM) at the mid-logarithmic phase in the absence of stress were mostly composed of polar lipids, indicating that the cell membrane is the likely origin of MEVs. A minor contribution of apolar lipids associated with the mycomembrane was observed (4). Whether those apolar lipids belong to MEVs originated at the cell membrane or to an independent population of MEVs linked to the mycomembrane was not investigated by then. In this context, a recent study on *Corynebacterium glutamicum*, a phylogenetically close species to *Mtb*, examined EV diversity upon exposure to genotoxic or cell wall stresses (33), suggests *C. glutamicum* undergoes bubbling cell death upon exposure to mitomycin-C releasing EVs. Moreover, when cell wall stress is induced by exposure to penicillin or biotin starvation, the release of EVs from the outer membrane is observed. These studies are important for understanding the universality of the general bacterial MVs biogenesis mechanisms. However, evidence of the formation of MEVs in *Mtb* when exposed to genotoxic stress has yet to be provided and some differences in the cell wall between *C. glutamicum* and *Mtb* raise questions about whether these mechanisms are conserved. For instance, differences in the length of the fatty acid portion of MA between *C. glutamicum* and *Mtb* (C32-36 vs C60-C90) may contribute to the differential release of these lipids. Another potential factor that may contribute to the release of other types of EVs is the time at which EVs are isolated, as EVs from the stationary phase may be released from cells whose bacterial viability is compromised. Therefore, this could favor explosive cell lysis.

Recent studies have shown that a cell wall imbalance in *Mtb* leads to an increase in MEV release (16, 19). For instance, the exposure of *Mtb* to sublethal concentrations of isoniazid (INH), a first-line antitubercular drug that targets mycolic acid synthesis, triggers MEV production (19). However, no lipidomic analyses were performed in this study. In an independent study focused on the functional analysis of *virR*, the first gene known to have a role in vesiculogenesis in *Mtb*, it was shown the absence of apolar lipids in MEVs derived from WT, a mutant in *virR* (*virR^mut^*) and a *virR* complemented strain when analyzed by thin-layer chromatography analysis or mass spectrometry (MS), suggesting that MEVs from *Mtb* do originate at the cell membrane under normal growing conditions (16). Acknowledging that some of the stresses analyzed in closely related species to *Mtb* may be physiologically relevant (33), we still lack confirmation that MEVs can include mycomembrane lipids and that their release is triggered by dying *Mtb* cells.

## GENETIC AND ENVIRONMENTAL FACTORS REGULATING MEV FORMATION

Bacterial vesiculogenesis is a sophisticated phenomenon in which a piece of membrane organizes itself in the form of vesicles that are released from the cell. In the case of E-type EVs, lipid fragments reorganize themselves to create EVs. Nevertheless, the fact that several compartments of the cell need to be modified to allow vesicle formation and release suggests that its regulation might be performed at multiple levels. One consequence is that no null mutant with a fully impaired ability to release EVs has been isolated to date. Seminal studies on Gram-negative bacteria either comparing the magnitude of EV production between single isogenic mutants with WT strains or by using forward genetic approaches screening for mutants with altered EV production (13, 14, 34), indicate that vesiculogenesis is not only dependent on a single gene. These studies revealed that up to 150 genes were involved in EV production in *E. coli* and that these genes are clustered in loci governing outer membrane components, peptidoglycan synthesis, and the σE cell envelope stress response (13). Another study in *Salmonella enterica* serovar Typhi searched for genetic determinants of the incorporation of toxins into EVs and found nine genes involved in envelope stability, accumulation of misfolded proteins, or lipopolysaccharide (LPS) composition (14).

There is a need for studies on genetic factors controlling Gram-positive bacterial EVs, and there are a few of them that examine EVs in isogenic mutants relative to WT counterparts. To date, evidence for the contribution of several transcription factors or two-component systems to EV production in unrelated Gram-positive bacteria has been provided, suggesting that their involvement may also be dependent on downstream genes (35, 36). Alternatively, phenol-soluble modulin (PSMs)-controlling genes have been shown to play a role in EV formation in *S. aureus* (30). The scenario in *Mtb* is not different from that in Gram-positive bacteria. So far, as few as three genetic determinants and several growing conditions have been shown to play a role in MEV production.

### *virR* as the first known *Mtb* genetic determinant of vesiculogenesis

*virR* was the first gene identified to have a role in MEVs. This gene was identified in a forward genetic screen using a transposon (Tn) library of more than 10,000 loss-of-function mutants as an endogenous regulator of *Mtb* immunostimulatory potential (17). It was shown that *virR* mutant (*virR^mut^*) augmented cytokine responses in mouse and human macrophages, concomitant with an attenuated phenotype in both experimental infection models. Subsequent studies established that *virR^mut^* overproduces MEVs, which contributes to enhanced host inflammatory or immune responses, providing a partial explanation for the observed attenuation (15). Of note, sequence analysis of the VirR protein indicates that it contains a conserved LytR C-terminal (LytR_C) domain, which is usually found in LytR-Cps2A-Psr (LCP) proteins. The LCP protein family includes enzymes that transfer glycopolymers from membrane-linked precursors to PG or cell envelope proteins and are central to cell envelope integrity in Gram-positive bacteria (37). *Mtb* has six genes encoding LCP proteins, three with both a catalytic LCP domain and LytR_C domain (Rv3267, Rv3484, and Rv0822), two with a single LytR_C domain (VirR and Rv2700), and a single LCP catalytic domain (Rv3840). In mycobacteria, some members of the LCP family of proteins have been implicated in the linkage between AG and PG (38, 39). Rv3267, Rv3484, and Rv0822 appear to have overlapping functions in cell wall assembly, with Rv3267 as the primary ligase (38). Interestingly, while knocking down the gene encoding the main AG-PG ligase (cg0847, LcpA) in *C. glutamicum* leads to the release of outer membrane material to the extracellular medium (40), this phenotype is not observed in any knockout strain in the *Mtb* orthologs (38), probably reflecting the ability of the mutants to negatively regulate the biosynthesis of cell wall constituents in response to a decrease in ligase activity. Nevertheless, the mechanism by which *virR^mut^*, defective in a LytR single domain protein with no catalytic domain, could generate a hypervesiculation phenotype was not known. A recent study investigating the mechanistic role of VirR in *Mtb* vesiculogenesis indicated that this protein may indirectly control cell permeability via PG remodeling (16). In this study, a detailed ultrastructural, transcriptional, proteomic, and lipidomic analysis of *virR^mut^* revealed profound cell wall alterations at the PG level. This mutant possesses an enlarged PG layer with a higher number of pores, but smaller than WT and complemented strains, as shown by cryo-electron microscopy (cryo-EM) analysis and high-resolution atomic force microscopy (AFM) of whole cells and purified PG, respectively (16). This phenotype is linked to the observed enhanced permeability of *virR^mut^* and the mutant in the homolog gene *cei* (cell envelope integrity, Rv2700) (16, 41), indicating that PG defects derived from the absence of VirR lead to enhanced permeability and vesiculation. These two processes seem to be connected since unrelated conditions that either induce or suppress permeability modulate EV levels in *Mtb* (16). The PG defects observed in *virR^mut^* may reflect an aberrant function of canonical LCP proteins since it was shown that VirR interacts with all LCP proteins via the LytR domain (16). VirR could, therefore, function as a scaffold for essential enzymatic functions in the cell wall of *Mtb*.

Although these findings shed new light on the biogenesis of MEVs, we still need to understand some aspects of this process. First, we do not know whether PG biosynthesis/degradation and vesiculogenesis are connected. In this context, it is possible that enhanced vesiculogenesis is a consequence of the cell envelope damage caused at different levels including membrane, AG-PG complex, or mycolic acids. Supporting this notion is the observed enhanced vesiculation in *Mtb* upon exposure to INH, a mycolic acid synthesis targeting drug (19). In this regard, the observed enhanced vesiculation in *virR^mut^* might be a consequence of the altered PG structure and the associated increased permeability. Therefore, vesiculogenesis could be a process that *Mtb* modulates to cope with cell envelope stress.

### Iron starvation stimulates vesiculation in *Mtb*

Like most living organisms, *Mtb* requires iron as a cofactor for vital enzymes (42). *Mtb* copes with nutritional immunity (43) inside the host by naturally activating a genetic program that includes genes involved in iron capture and transport (42). Some of these genes are required for virulence, indicating that iron availability is linked with bacterial proliferation in the host. Notably, when *Mtb* is cultured under iron starvation conditions, it responds by increasing MEV levels (24), supporting the notion that vesiculogenesis is important for the survival strategy of the bacillus. Lipidomic analysis of low-iron MEVs showed that they are mostly composed of cell membrane lipids and the presence of iron-loaded siderophores, indicating that MEVs serve as a vehicle to capture iron. Notably, evidence that MEVs may act as iron carriers was demonstrated in growth restoration experiments of *Mtb* mutants deficient in siderophore synthesis, which are unable to grow in low-iron medium when such cultures were supplemented with low-iron MEVs (24). How iron starvation leads to enhanced vesiculation is still an unanswered question. It is possible that the large amounts of lipidic siderophores produced under iron starvation that accumulate in the cell wall can interact with cell membranes inducing membrane blebbing. This phenomenon has been observed in two iron-starved Gram-negative bacteria like *Haemophilus influenzae* and *Vibrio cholerae*, where phospholipid accumulation in the outer leaflet of the outer membrane leads to enhanced (44).

### Dynamin-like proteins IniAC

Common transcriptional signatures between two unrelated conditions leading to enhanced vesiculation including *virR^mut^* and iron starvation revealed the overexpression of the *iniBAC* operon (19). This operon is induced in *Mtb* upon exposure to cell wall-targeting drugs such as INH, via activation of the upstream transcriptional regulator IniR (45, 46). The transcriptional activation of *iniBAC* is mediated by IniR after the sensing of free trehalose upon treatment with INH (46). A recent report identified *iniA* and *iniC* as dynamin-like proteins that are involved in MEV production (19). Dynamins are large GTPases that can promote fusion or fission of membranes in eukaryotic and prokaryotic cells (47). It was demonstrated that the inactivation of *iniAC* in *Mtb* reduced MEV levels and restoration of WT MEV levels required both genes, suggesting potential cooperation of IniA and IniC (19). Accordingly, when *iniAC* was either genetically or chemically (via sublethal treatment with INH) overexpressed in *Mtb*, enhanced MEVs were observed concomitant to an increase in membrane curvature events as observed by cryo-EM (19). This phenomenon was also observed in a *Mycobacterium smegmatis* (*Msmeg*) strain overexpressing *Msmeg* IniA. This study demonstrated a GTP-dependent membrane fission activity of IniA *in vitro* (48). It is therefore tempting to speculate that both IniA and IniC work together to assist mycobacterial cell membrane remodeling to allow the release of MEVs.

### Pst/SenX3-RegX3

EV release in Gram-negative bacteria has been suggested to represent a type 0 secretion system (T0SS), taking into consideration the similarities with classical secretion systems and the fact that EVs can be highly effective in transporting lipids, hydrophobic molecules, and virulence factors in a concentrated manner (5). Besides canonical Tat and Sec secretion systems, *Mtb* has five specialized type VII (T7SS) secretion systems, collectively referred to as the ESX systems, that have been shown to play an important role in pathogenesis (3). Among ESX systems, ESX-5 is known for its role in inducing inflammation and caspase-dependent cell death (49). Moreover, ESX-5 is regulated by the two-component system SenX3-RegX3 in response to phosphate starvation (50). Mechanistically, inhibition or activation of the SenX3-RegX3 system is mediated by the direct interaction with a phosphate transport system (Pst), which senses phosphate. The DNA binding response regulator RegX3 transcriptionally controls not only the expression of genes involved in phosphate scavenging but also *esx-5* genes (51). Interestingly, a mutant defective in *pstA1*, a component of the phosphate transport system, exhibits an enhanced transcription of esx-5 genes and hypersecretion of ESX-5 substrates as well as LpqH and PstS1 lipoproteins, all of them previously reported as MEV components by several proteomic reports (4, 11, 52). Based on these connections, investigations on the role of the Pst/SenX3-RegX3 have directly implicated this system in vesiculation in *Mtb*. For example, genetic disruption of *pstA1* caused a hypervesiculating phenotype, which is independent of ESX-5 activity and VirR, suggesting a novel mechanism of vesiculogenesis in *Mtb* (53).

## MEV CARGO

Irrespective of their mechanism of biogenesis, bacterial EVs can package a distinct set of biomolecules including proteins, lipids, DNA, and RNA, acting as a vehicle to transport these molecules out of the cell, to host cells or other bacteria. Therefore, compositional analysis of EVs is usually performed to better understand their physiological and biological function.

### Proteins

The first study on mycobacterial EVs did not provide any high-content proteomic analysis but showed that *M. ulcerans* EVs were loaded with mycolactone. *M. ulcerans* EVs containing mycolactone augmented their toxicity relative to purified mycolactone *in vitro* and *in vivo* (25, 54). Subsequent proteomic studies on *Mtb*, BCG, and *Msmeg* identified 48, 66, and 64 MEV-associated proteins, respectively (4). Further proteomic analysis of MEVs using more sensitive MS equipment indicated a global proteome of 200 proteins, validating the previous analysis (52). Of note, *Mtb* and BCG MEVs were enriched in lipoproteins and proteins implicated in cell wall processes, membrane biology, and intermediate metabolism (4, 52). Conversely, *Msmeg* EVs did not show significant enrichment for lipoproteins (4), suggesting a differential incorporation of proteins into MEVs between species. Remarkably, it is known that the proteome of Gram-negative MVs is subjected to change according to a range of factors including antibiotic stress (55) or nutrient availability (56). This supports the notion that bacteria tailor MV cargo in response to environmental cues. In this context, it has been shown that submitting mycobacteria to stresses that seek to reproduce the intraphagosomal environment, such as iron starvation or low pH, changes the protein composition of EVs (11, 57). A recent study on the serological responses of TB patients to low-iron and high-iron MEVs, which included a proteomic analysis of these two sets of MEVs, revealed that *Mtb* reduces MEVs protein cargo under iron starvation but preserves most prevalent proteins, such as lipoproteins (11).

### Lipids

Lipidomic analysis by thin-layer chromatography (TLC) of MEVs isolated from *Mtb* MM mid-log phase cultures revealed a MEV lipid composition consisting mainly of phospholipids [phosphatidylglycerol (PG), phosphatidylethanolamine (PE), phosphatidylinositol (PI) and cardiolipin], acylated phosphatidylinositol dimannosides and hexamannosides (PIM_2_ and PIM_6_) and, in trace amounts, polyacylated trehalose and phenolic glycolipids (4), indicating an origin linked to the cell membrane. The presence of polar lipids and some minor acylated glycerides and PE was detected in low-iron MEVs along with iron-loaded siderophores in comparison with MEVs produced by iron-sufficient *Mtb* (24). While a more detailed analysis of MEVs based on high throughput MS is currently lacking, TLC analysis indicates that polar lipids represent the major component of MEVs with lower amounts of apolar cell wall-associated lipids also detected. To understand whether such apolar lipids are also an integral part of MEVs or they represent an independent population of EVs more investigations are needed. A recent study on *C. glutamicum* EVs isolated from cultures submitted to cell wall stresses including penicillin G treatment or biotin starvation, identified the outer membrane as the origin of EVs (33). Whether this mechanism, which leads to the biogenesis of cell wall-derived EVs, is also conserved in *Mtb* awaits experimental confirmation.

### Nucleic acids

Dorward et al. reported the presence of DNA and RNA in EVs from *E. coli* 30 years ago (27). Since then, the detection of nucleic acids in bacterial EVs has been widely reported (58, 59) and, therefore, it is likely that MEVs also contain nucleic acids including DNA and RNA. Although DNA in MEVs has never been confirmed, double-stranded DNA (dsDNA) has been found in *Mycobacterium avium*-derived EVs (57). Notably, another study on the RNA profiling of exosomes (EVs from eukaryotic cells derived from the endosomal pathway (60)) from *Mtb-*infected murine macrophages detected RNA of bacterial origin (61). Whether its presence is solely linked to an MEV population co-isolated with exosomes, incorporated into both exosomes and MEVs or becomes part exclusively of the exosome population needs further exhaustive investigation. Nevertheless, it remains to be elucidated how *Mtb* RNA gets incorporated into MVs. That MEVs carry ssRNAs has recently been indirectly demonstrated by showing how isolated MEVs can trigger intracellular toll-like receptor signaling *in vitro* and *in vivo* (21). These studies encourage further definition of RNA pools linked to MEVs and open a new field to investigate the biology of RNA transfer into MEVs and its connection with pathogenesis mechanisms.

### Lipoglycans

One of the most important molecules involved in the pathobiology of *Mtb* is lipoarabinomannan (LAM), an abundant surface-exposed, phosphatidylinositol (PI)-anchored lipoglycan with diverse biological activities, including mediating human cell entry, inhibition of phagosome-lysosome fusion, and intracellular survival (62, 63). Seminal studies focused on understanding whether major cell envelope-associated polysaccharides like LAM or a-glucan (32) are bonafide components of MEVs revealed that LAM can be isolated in MEVs along with lipoproteins, indicating that LAM is an integral part of MEVs (4). Taking into consideration that LAM can be either anchored to the cell’s inner membrane and the outermost mycomembrane via its PI moiety (64), it is tempting to speculate that MEVs originated at both locations may contain LAM. Acknowledging the immunomodulatory role of LAM and its presence in MEVs indicated that MEVs could play a role in the bacillus intracellular survival and persistence (4, 7, 8, 65).

## MEVs AT THE HOST-PATHOGEN INTERFACE

Initial studies focused on understanding the role of MEVs in host-pathogen interactions which included the isolation of MEVs from axenic cultures and the analysis of the immune response after vesicle stimulation. Such studies provided evidence for the immunomodulatory properties of MEVs. In fact, the first report on *M. ulcerans* EVs demonstrated their cytotoxicity on both phagocytic and non-phagocytic cells since these EVs included the toxin mycolactone (25). Further studies on *M. ulcerans* MEVs showed that this toxicity leads to the induction of interleukin 1β (IL-1β) production in human macrophages and a strong inflammatory response in mice (54). The appreciation that pathogenic bacterial species tend to concentrate virulence factors in EVs is also true for *Mtb*. Specifically, it was shown that stimulation of murine macrophages with MEVs promoted a pro-inflammatory response mediated by toll-like receptor 2 (TLR-2) because of their enrichment in lipoproteins and other TLR-2 agonists (4). This response could also be reproduced *in vivo* when MEVs were administered intratracheally prior to a challenge with virulence mycobacteria. A profound granulomatous inflammation and higher bacterial loads in lungs and spleens were observed in WT mice but absent in TLR-2^−/−^ mice. Notably, such gross effects of MEVs *in vivo* were not observed when *Msmeg*-derived EVs were similarly administered (4), suggesting that differential MEV composition between *Mtb* and *Msmeg* determines its immunomodulatory properties.

At present, there is not a clear picture of how MEVs are produced and released during an ongoing infection. Early reports on EVs in the context of mycobacterial infections provided evidence that exosomes isolated from *Mtb*-, BCG, or *M. avium*-infected macrophages possess strong immunomodulatory properties due to the inclusion of mycobacterial antigens (66), albeit a marginal contribution to the pool of peptides identified by MS. Although the concept of MEVs was not considered at that time, it was clear that mycobacterial antigens could be exported out of the infection site via EVs. Subsequent studies showed that MEVs and exosomes can represent two independent vesicle populations in the context of an *in vitro* macrophage infection (65). Both populations were separated based on either *Mtb* markers including LAM or LpqH, or exosome markers like CD9, CD63, or MHCII (65). Importantly, this study emphasizes that MEVs might have been overlooked in studies focused solely on exosomes. Nevertheless, a molecular definition of both EV populations in terms of protein and lipid composition has never been provided. Such studies would help to better understand the interplay between *Mtb* and the macrophage. In any case, all those studies provided evidence for the fact that MEVs are promoters of inflammation and that such responses may be detrimental to the host. In a different line of research also critical for an antimycobacterial immune response such as the antigen presentation process, it was shown that MEVs could promote the inhibition of antigen presentation by impairing interferon (IFN) mediated expression of major histocompatibility complex (MHC) II molecules in naïve macrophages (7). Similarly, it was shown that MEVs inhibited IL-2 production and reduced T-cell proliferation following TCR stimulation (8). Conversely, maturation and enhanced antigen presentation to antigen-specific CD4^+^T cells by MEV-treated dendritic cells was shown in a subsequent study (67). This apparent contradiction may be explained by the temporal dependence of TLR-2 stimulation of antigen-presenting cells (68, 69) since sustained TLR-2 stimulation reduces MHC-II expression leading to the blocking of IFN response (69). Definitive proof that MEVs critically contribute to modulate the immune response during an *in vitro* infection was partially provided in a recent study where the isolation of a *Mtb* mutant in the dynamin-like proteins *iniAC*, deficient in MEV production, showed that, albeit having similar intracellular replication rates than the WT counterpart in macrophages, the stimulatory properties of the Δ*iniAC*-infected macrophage supernatant on naïve macrophages were strongly reduced (19). This suggests that MEVs critically contribute to modulating the immune environment of the infecting site.

It seems that TLR-2 is not the only receptor capable of sensing MEVs. In a recent study, *Mtb* replication was examined in the context of infection of THP-1 macrophages, transduced with a genome-wide CRISPR library, to identify novel host restriction factors (21). This study focused on TLR-8, as it is known to be involved in the xenobiotic response and is within the druggable genome (70). TLR-8 is a known endosomal sensor of ssRNA and an established mediator of antiviral immunity (71). A mechanistic analysis of TLR-8’s role during *Mtb* infection revealed that it is activated by phagosomal mycobacterial RNA released by MEVs and leads to enhanced xenophagy-dependent *Mtb* killing (21). Although intracellular TLR sensing by nucleic acid-loaded bacterial MVs has previously been reported for other unrelated bacteria (58, 72–74), these results open a new field in *Mtb* physiology and encourage to investigate how RNA gets incorporated into MEVs as well as how RNA pool may change according to the conditions faced by the bacillus.

## MEVs AS PROMISING PLATFORMS TO DEVELOP NOVEL ANTI-TB TOOLS

There are many properties of bacterial EVs that make them ideal platforms for biomedical applications. In the context of TB, areas including vaccine development, treatment, and diagnosis are in urgent need of novel approaches that help to tackle the disease.

## MEVs AS AN ALTERNATIVE VACCINE

As bacterial MEVs contain bacterial derived antigens and multiple pathogen-associated molecular patterns (PAMPs) they may represent a new alternative to current vaccines. Indeed, bacterial EVs from several Gram-negative bacteria elicit strong both humoral and cellular immune responses when given to mice (75). The approval of a vaccine against *Neisseria* based on OMVs demonstrated the potential of bacterial MEVs to be clear alternative vaccines (75, 76). These properties are also applicable to Gram-positive bacteria EVs as demonstrated by the protection provided by *Streptococcus pneumoniae* EVs when administered to mice prior to an infection with a virulent strain (77). As for *Mtb*, it was demonstrated that subcutaneous administration of MEVs to mice prior to a challenge with a low dose of virulent *Mtb* showed protection by MEVs at a similar level to standard BCG vaccination, in the form of control of bacterial replication in lungs and spleens and reduced lung inflammation (9). Of note, the induction of MEV-associated protective responses was achieved with no adjuvants indicating that MEVs also contain potent PAMPS that may help to enhance and modulate immune responses to specific antigens, a property also common to other bacterial EVs preparations (78). Although most of the measured humoral and cellular immune responses upon MEV immunization were shown to be directed to cell surface antigens, including lipoproteins, it remains to be determined what part of those antigens are responsible for the protective responses. Indeed, MEV biogenesis is a stochastic phenomenon that may lead to composition heterogeneity of MEVs that eventually may provide differential degrees of protection, as was previously shown (79). The definitive answer to the potential of MEVs as an antituberculosis vaccine would include the generation of defined artificial MEVs including MEV-associated antigens (protein, lipids, nucleic acids) with protective and adjuvant properties.

## MEVs AS THE CENTRAL CORE OF NOVEL DIAGNOSTIC APPROACHES

An EV is a biological particle containing information about the cell that released it. This notion is particularly important for a cryptic microorganism like *Mtb*, whose physiology cannot be separated from that of its only host, the human. Most of *Mtb*’s biology occurs inside host cells and MEVs derived from this interaction can provide valuable information about the status of the bacterium. Proof that MEVs could represent a novel concept with potential applications to TB diagnostics has been shown in the form of indirect and direct approaches. The first report showing that MEVs contain biomarkers with diagnostic potential in serology included a small cohort of individuals with smear-positive, or negative TB infection, and BCG-vaccinated with and without latent TB. The recognition of a pool of three MEV-antigens discriminated between TB+ and non-TB individuals (10). A more recent study aimed to compare the immunogenicity of MEVs isolated from normal and low-iron *Mtb* cultures similarly. This study provided also the proteomic composition of both sets of MEVs and showed that iron starvation, albeit triggering MEV production in *Mtb* (24), reduces the number of proteins loaded into MEVs (11). Notably, the most abundant antigens detected in MEVs did not change in either condition, suggesting that they may represent bonafide MEV markers. The serology study included a cohort of 90 individuals from Barcelona (Spain) distributed in 30 healthy controls, 30 latent TB-infected (LTBI) individuals, and 30 active TB individuals. Results showed that low-iron MEVs by themselves may be used to discriminate TB from non-TB individuals. Moreover, the combination of three MEV-associated proteins has almost perfect features to discriminate TB from non-TB individuals (11). The fact that both studies were carried out in two independent cohorts supports studying the feasibility of this antigen pool in a different and bigger cohort.

A recent report provided proof for the detection of MEV in plasma in children with HIV, a population that is often missed by sputum-based diagnostics (80). Authors develop a system based on dark-field microscopy supported by machine learning focused on the capture and detection of circulating MEVs via LAM and the lipoprotein LprG, another major MEV-associated antigen (9). This assay could distinguish active TB from LTBI in non-human primates and, more importantly, it detected 74% of cases of pediatric TB, 73% of cases missed by microbiological assays, and 80% of cases missed during the study in a cohort of 147 children (80). Acknowledging the difficulties associated with samples from pediatric populations, these results open a new avenue to develop TB diagnostic assays based on MEVs in a point-of-care format. In addition, it supports further research on the adult TB population and the use of alternative fluidic samples including urine.

## CONCLUDING REMARKS

The new paradigm in cell-cell communication involving the trafficking of EVs has revolutionized many of our concepts of cellular physiology. The realization that cell-walled bacteria such as mycobacteria and Gram-positives also make EVs has only taken root in the past decade. The major hurdle when considering EV production by cell-walled microbes is the belief that the cell wall is a rigid structure that prevents vesicular transit. Moreover, explaining EV release becomes a more difficult task for bacteria with unconventional cell envelopes such as *Mtb*. Vesiculation is also universal in mycobacteria and accumulated evidence suggests that *Mtb* uses MEVs as part of its sophisticated strategy to subvert the immune system. However, evidence also suggests that MEVs are a good source of biomarkers, and they might be good surrogates of the infection status. In addition, MEVs have been demonstrated to have good adjuvant and vaccine properties.

Despite the accumulated knowledge on MEVs in the past 15 years, there are some gaps and questions that need clarification. For instance, contradictory reports are indicating the distinct origins of MEVs either from cell membrane or mycomembrane, which might reflect the use of different strains or simply different time points at which EVs are isolated. Microscopy data show MEVs as either unilamellar or bilayered which indicates different lipid composition and potentially different biogenesis pathways. In addition, improvement in the separation of MEVs according to validated markers across strains and growing conditions would help to clarify these findings. We also need more information about the genetic determinants of vesiculation in *Mtb*. Screening for mutants with altered vesicle production would provide valuable information about the pathways implicated in this process.

Importantly, an integrative proteomic approach including (i) MEVs from axenic cultures submitted to different growing conditions, (ii) MEVs isolated from *Mtb*-infected macrophages, (iii) MEVs isolated from plasma of *Mtb*-infected mice, and (iv) MEVs isolated from human fluidic samples (plasma and urine) from TB individuals would provide critical information about the heterogeneity of EVs in the context of *Mtb* infection at many different levels.

Another major gap in the field is to understand how MEVs can exit infected macrophages. During this trajectory, it is not clear whether MEVs interact with the endocytic network, or they simply reach the extracellular space by themselves, or both. Fluorescence-based approaches including markers of both MEVs and exosomes could help to track the fate of EVs containing *Mtb* antigens during an ongoing infection.

To properly determine the real potential of MEVs as a vaccine or vaccine adjuvant, approaches will need to focus on generating artificial EVs containing protective antigens (protein and lipids) that should be initially tested in preclinical models of infection. Finally, TB diagnostics could greatly benefit from the use of MEVs as either a source of biomarkers or by the direct detection of fluidic samples. Taking all together, MEVs could represent a new paradigm in the development of novel tools to tackle TB.
